# Institutional Surgical Setting and Volume Effects of Certified Arthroplasty Centers in Germany: Evaluation of the Quality of Care in a 5-Year Comparison

**DOI:** 10.3390/healthcare12090904

**Published:** 2024-04-26

**Authors:** Katrin Osmanski-Zenk, Annett Klinder, Andreas Pingsmann, Christoph H. Lohmann, Hermann Josef Bail, Bernd Kladny, Wolfram Mittelmeier

**Affiliations:** 1 Orthopedic Clinic and Policlinic, University Rostock Medical Center, 18057 Rostock, Germany; annett.klinder@med.uni-rostock.de (A.K.); wolfram.mittelmeier@med.uni-rostock.de (W.M.); 2 Professional Association for Orthopaedics and Trauma Surgery, Biberburg Orthopaedic Associates, 14089 Berlin, Germany; a.pingsmann@biberburg.de; 3 Department of Orthopaedic Surgery, Otto-von-Guericke-University, 39120 Magdeburg, Germany; christoph.lohmann@med.ovgu.de; 4 Department of Orthopedics and Traumatology, Paracelsus Medical University, 90471 Nuremberg, Germany; hermann-josef.bail@klinikum-nuernberg.de; 5 Fachklinik Herzogenaurach, D-91074 Herzogenaurach, Germany; bernd.kladny@fachklinik-herzogenaurach.de

**Keywords:** EndoCert, quality assurance, senior/EndoCert-registered surgeon, affiliated surgeon, volume effects

## Abstract

To improve arthroplasty care quality, the EndoCert initiative focuses on structural, processual, and surgeon-related quality assurance. The aim of this study was to assess the impact of a surgeon’s case load in certified centers on quality of care, distinguished by different types of surgeons. Data from the annual reports of EndoCert certified centers for the years 2017 to 2021 were analyzed. The study revealed reduced numbers of cases, while the number of surgeons remained constant. Since 2020, the decrease in the average case load per surgeons has become more pronounced. There were also differences between senior (sECrs) and EndoCert-registered surgeons (ECrs). Before the 2020 pandemic, over half of surgeons exceeded minimum annual case requirements, while, afterwards, this number declined, especially for the ECrs. Affiliated surgeons, who are also sECrs or ECrs, performed predominantly lower numbers of arthroplasties. However, a higher percentage of affiliated surgeons in a center correlated with faster surgeries and lower mortality rates. High numbers of arthroplasties per center or surgeon were not necessarily associated with better quality indicators, especially in the knee. While the comprehensive quality standards may offset volume effects, EndoCert should reconsider minimum volume regulations based on surgeon, but also on each joint.

## 1. Introduction

The EndoCert initiative was established by the German Society for Orthopedics and Orthopedic Surgery (DGOOC) to ensure quality in arthroplasty care. This initiative has now been in use for more than 10 years [1]. There is no other comparable quality assurance system which has been internationally established in the field of total joint arthroplasty. The main pillars of the EndoCert certification process are requirements for structural and process quality, but also center- and, above all, surgeon-related volume regulations, which are monitored during the annual inspections of the centers by independent experts [2,3].

Patient-specific characteristics such as body mass index, and comorbidities, but also socio-economic and psychological factors, determine the peri- and postoperative outcome after joint replacement surgery [4]. Clinical, surgical, and implant-specific outcome measures can counteract negative influences due to these factors [5]. Furthermore, the surgeon plays a significant role. It is well-known that a surgeon’s experience throughout the entire treatment process plays a pivotal role in the treatment outcome. The expertise depends not only on the total number of performed arthroplasty procedures, but also on the number of arthroplasty procedures conducted on a regular, annual basis [1,5,6,7,8,9,10].

Since its launch in 2012, the EndoCert initiative aimed to introduce and establish various quality assurance measures at a structural and procedural level. These consider the abovementioned measures to enable the control of influencing factors. A key element of this concept is the requirement that hip and knee arthroplasties are exclusively performed by EndoCert-registered surgeons (at least 50 total joint arthroplasty (TJA)/year) or senior EndoCert-registered surgeons (at least 100 TJA/year). However, it should be noted that the surgeon-related minimum volumes comprise hip as well as knee arthroplasties [1,2,3].

The aim of this study was to investigate the impact of volume distribution and effects in relation to the number of cases of senior EndoCert-registered surgeons (sECrs) and EndoCert-registered surgeons (ECrs). Therefore, this article answers the question of how different organizational settings of surgeons in a certified center, such as the presence of affiliated surgeons, affect the quality of care which was measured on the basis of center-specific quality indicators. In a 5-year comparison, the effects of the changes of care structures due to the introduction of the EndoCert certification system were evaluated for the first time. The novelty of this study is to evaluate how different institutional settings of surgeons in certified arthroplasty centers that have implemented various quality assurance measures affect quality of care, as measured by center-specific indicators. Previous research [11,12,13] has examined the general relationship between surgeon volume and clinical outcomes. This provides insights into how volume-based regulations and quality programs can influence outcomes in a certified care setting.

### Background

Prior to the launch of the EndoCert initiative in October 2012, the certification system was simulated and tested in two pilot phases, first in 10, and then in 13 centers [14].

Currently (as of January 2024), 495 arthroplasty centers (AC) or maximum-care arthroplasty centers (ACmax) are certified. Participation in this certification system is voluntary; there is no legal obligation to undergo this certification process in order to perform arthroplasties. According to the German Hospital Directory, 1136 clinics in Germany currently provide patients with TJA. According to this, the proportion of EndoCert centers is around 44% [15].

According to the Federal Statistical Office, a total of 460,763 TJA (OPS coding 5-820-5-823) were treated nationwide in 2021. In the same year, 250,580 TJA were registered in the EndoCert system (excluding tumor TJA), meaning that more than 54% of all TJA in Germany are performed within the EndoCert system [16].

In 2021, a total of 1647 designated EndoCert-registered surgeons (ECrs) and 529 senior EndoCert-registered surgeons (sECrs) were active in the system. According to the German Medical Association’s physician statistics as of 31 December 2021, 981 surgeons were working in “Orthopedics” and 8243 in “Orthopedics & Trauma Surgery” in a hospital environment. According to these statistics, there were also 710 hospital doctors with the additional qualification “Special Orthopedic Surgery” (SOC). In 2021, 619 surgeons with the additional qualification SOC were registered in the EndoCert system, representing over 87% of surgeons with this qualification [17].

The prerequisite for approval as a senior/EndoCert-registered surgeon and maintenance of status is as follows:

The following requirements must be met in order to be approved as a senior/EndoCert-registered surgeon:

1. Proof of case volume: 200 (sECrs) or 100 (ECrs) of TJA with main responsibility (as primary surgeon, mandatory assistance by s/ECrs) performed over a minimum of 12 and a maximum of 24 months at the registered AC/ACmax;
2. Specialist in orthopedics and trauma surgery;
3. sECrs can only operate in an ACmax. Even if an ECrs in an AC meets the requirements, as mentioned above, for a sECrs, an upgrade to sECrs is not possible.

To maintain this status after approval, the following has to be provided:

1. Evidence of at least 50 (ECrs) or 100 (sECrs) arthroplasty procedures from TKA and THA (including revision surgery) per year at the center. The minimum volume refers to all performed or assisted surgeries;
2. Proof of attendance of advanced training courses in the field of arthroplasty (at least three courses within 3 years) [2,3];
3. In addition, s/ECrs (senior EndoCert-registered surgeons and EndoCert-registered surgeons) must perform the majority (>50%) of their arthroplasty surgeries at the certified center. Depending on the type of center (AC vs. ACmax), one of the s/ECrs must have the additional qualification “Special Orthopedic Surgery”. Since 2021, it has been possible for s/ECrs to work at two ACs/ACmaxes and for the procedures to be allocated to the respective centers [18]. Klicken oder tippen Sie hier, um Text einzugeben.

It should be noted that the designation of s/ECrs is an EndoCert-initiative-specific qualification, while SOC represents an independent supplementary training to qualify for the treatment of more complex issues in congenital and acquired diseases or deformities of the musculoskeletal system.

Generally, affiliated surgeons from local practices, who are not employed by an AC/ACmax, may also qualify as an s/ECrs according to the mentioned prerequisites. They are entitled to treat their patients in the AC/ACmax and to use its infrastructure.

## 2. Materials and Methods

The study was based on the annually provided data of the certified centers to the external independent accredited certification body. Appropriate approval to conduct the study was obtained from the local ethics committee (file number: A2023–0114) on 12 July 2023 and consent was obtained from the centers. 

All ACs/ACmaxes that were certified according to the EndoCert procedure in Germany in 2017 (n = 539), 2018 (n = 526), 2019 (n = 515), 2020 (n = 495), and 2021 (n = 480) were included. Furthermore, all s/ECrs that were registered in the respective data year were included in this study. This specification resulted in the fact that s/ECrs who did not perform any TJA in a data year and those who performed fewer than 50 TJA per year were also included in the study. The reasons for this deviation are listed below:

1. Reasons for s/ECrs having zero cases per year:
  - s/ECrs left the AC/ACmax at the beginning of the year;
  - s/ECrs was subsequently registered at the AC/ACmax at the end of the year.
2. Reasons for s/ECrs having fewer than the total number of 50 cases per year:
  - s/ECrs left the AC/ACmax during the year;
  - s/ECrs was subsequently registered during the year;
  - s/ECrs fell ill/maternity leave/parental leave;
  - s/ECrs did not meet requirements;
  - Pandemic-related reduction in the number of cases in 2020 and 2021.

Since each named s/ECrs provided proof of qualification over a number of years, the authors assumed that the reduced minimum volumes described above have no influence on the results and were, therefore, included as specified.

Data were analyzed related to the AC/ACmax per data year and to the respective s/ECrs per data year. In addition, six case volume groups (0–49, 50–75, 76–100, 101–150, 151–200, >200 cases per surgeon) were defined to further classify the surgeons’ proficiency. 

Since the total number of cases per surgeon can vary within the centers, while the quality indicators (QIs) always refer to a center as an entity, a so-called surgeon ratio per center was defined. The surgeon ratio was calculated as follows:

Surgeon ratio = number of all TJA per center/sum of all s/ECrs per center

Accordingly, a high ratio means that more TJA per surgeon were performed at the center. The implementation of the ratio was intended to answer the question of the extent to which the total case volume of the s/ECrs affects the frequency of the respective center-related quality indicators.

In the German healthcare system, surgeons do not have to be employed by the hospital in which they operate on their patients. The affiliated surgeons from local private practices may use the infrastructure of the AC/ACmax and provide their patients with surgical care in these centers via the “affiliated physician system”. A rate was defined in order to measure the quality of this form of organizational arrangement compared to centers with only in-house surgeons:

Affiliated surgeon rate/ratio = number of affiliated surgeons per center/sum of all S-/PO per center × 100

Accordingly, a high affiliated surgeon rate means that more affiliated surgeons are part of the AC/ACmax.

### Statistical Analysis

The statistical analysis was performed using SPSS Statistics 27.0 software (IBM Corp., New York, NY, USA). Data were calculated separately for each data year (n = 5) for the years 2017 to 2021. Additionally, analyses were performed independently for primary joint arthroplasty, revision surgery, and fracture-related arthroplasty.

For continuous variables, the mean values (mean), minimum (min), maximum (max), sum (Σ), and number of available observations (n) were calculated. For categorical variables, the absolute and relative frequencies were determined. A normal distribution of continuous variables was analyzed with the Kolmogorov–Smirnoff test. Since the data were not normally distributed, non-parametric tests were used in the subsequent analyses. To assess the strength of the relationship between two continuous variables, the correlation coefficient (r) according to Spearman was computed. The *p*-value and the number of variable pairs were reported. Cohen’s classification (1992) [19,20] was utilized for effect size assessment, categorizing r ≥ 0.10 as a weak effect, r ≥ 0.30 as a moderate effect, and r ≥ 0.50 as a strong effect. For the comparisons of categorical variables, cross-tabulations were used, including a chi-square test to evaluate significant differences between groups. A z-test, with Bonferroni correction, was used to compare column proportions. In general, *p* < 0.05 was considered statistically significant.

## 3. Results

### 3.1. Center-Related Results

#### 3.1.1. Number of EndoCert-Registered Surgeons and Senior EndoCert-Registered Surgeons Working in the Participating Centers in the Years 2017–2021

Table 1 gives an overview of the number of certified ACs/ACmaxes for the respective data years and the center-related total numbers of the respective types of surgeon. This shows how many s/ECrs, affiliated s/ECrs, and s/ECrs with SOC registered at ACs/ACmaxes.

The number of certified ACs/ACmaxes decreased over time, while the total number of s/ECrs remained constant (Table 1). The number of affiliated s/ECrs also remained constant over time. The number of s/ECrs with SOC increased significantly over time (+13%).

#### 3.1.2. Total Volume of Cases Treated in Certified Centers per Year

**Figure 1:**
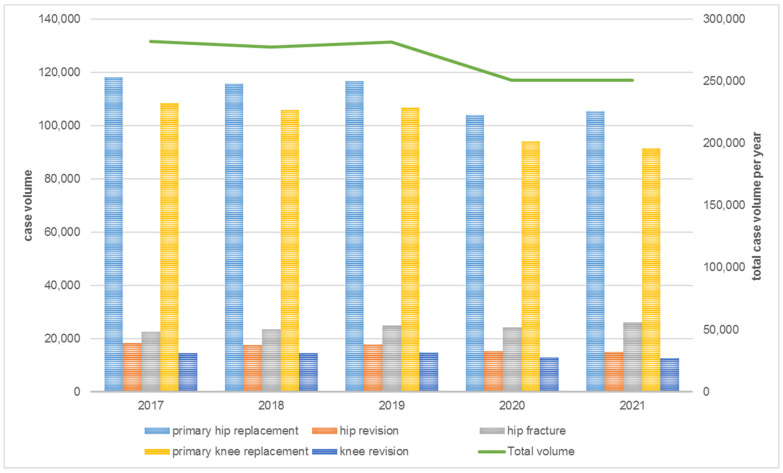
Center-related development of case volume in a year-on-year comparison in 2017–2021.

While the number of s/ECrs remained constant over time (Table 1), the number of cases declined (10–15%) for both primary joint arthroplasty and revision surgery, due to the pandemic. Only in hip fracture treatment did the volume of cases increase by 15% in 2021 compared to 2017 (Figure 1).

The constant number of s/ECrs (2017: n = 2177 vs. 2021: n = 2176) in combination with the decrease in the volume of cases in the EndoCert system over time means that since 2020 surgeons had a lower case load.

### 3.2. Surgeon-Related Results

#### 3.2.1. Volume Groups of Surgeons Differentiated by Registered Level of Experience (ECrs vs. sECrs) per Year

Table 2 shows the number and percentage of surgeons in the defined volume groups in total and differentiated by senior and EndoCert-registered surgeon, thus allowing a comparison between the registered levels of experience. The chi-square test showed that there were significant differences between all groups (*p* < 0.001). 

In the smaller volume groups, the number of ECrs is significantly higher than that of sECrs, and, in the largest volume groups, the number of sECrs is significantly higher than that of ECrs. For the ECrs, the highest percentage of surgeons achieved the volume group of 50–75 cases, whereas most of the sECrs operate more than 200 cases. In addition, the percentage of surgeons in the smaller volume groups increased over time, while, in the large volume, groups the percentage decreased.

Until the outbreak of the pandemic in 2020, more than half of all surgeons had treated significantly more cases than the minimum requirement of 50 or 100 TJA per year, depending on the registered level of experience.

#### 3.2.2. Volume Groups of Surgeons Differentiated by Affiliated Surgeons per Year

**Figure 2:**
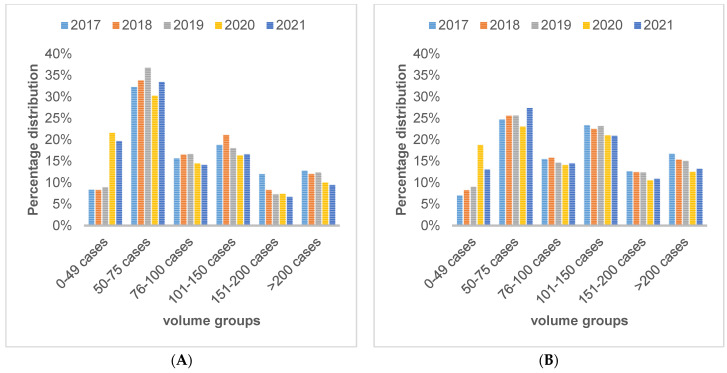
Percentage distribution of affiliated surgeons (**A**) and in-house surgeons (**B**) per volume group in a year-on-year comparison.

This 5-year comparison (Figure 2A,B) shows the percentage distribution of affiliated surgeons and in-house surgeons in each volume group of the centers. The majority of affiliated surgeons belonged in the smaller volume groups.

#### 3.2.3. Volume Groups of Surgeons Differentiated by Additional Qualification “Special Orthopedic Surgery” (SOC) per Year

**Figure 3:**
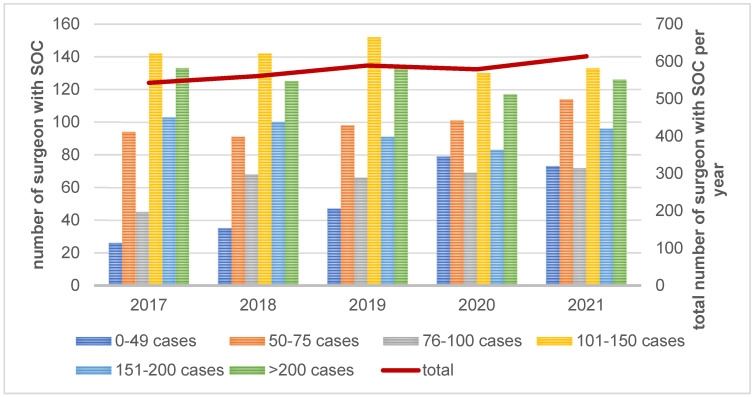
Distribution of surgeons with SOC per volume group in a year-on-year comparison.

Figure 3 shows a significant increase in the number of surgeons with SOC in the certified centers over time.

#### 3.2.4. Effects of the Surgeon Ratio on Frequency of the Quality Indicators

To answer the question of whether the defined surgeon ratio per center had an effect on the frequency of the respective quality indicators (QIs), a non-parametric correlation analysis was performed between the surgeon ratio and the percentages of the QIs.

In Table 3 below, the bold r- and *p*-values indicate a negative correlation, meaning that fewer TJA per s/ECrs per center results in a higher complication rate. The results in italics, on the other hand, show that the s/ECrs with higher case volumes per center have a higher complication rate. Only statistically significant results are presented.

All of the observed correlations were weak with r ≤ 0.30. For hip arthroplasty, there were predominantly negative correlations, i.e., the risk of complications in a center decreased when, on average, more surgeries per s/ECrs were performed in that center. In contrast, the results for knee arthroplasty showed that, except for operating time, a lower surgeon ratio was associated with fewer complications.

#### 3.2.5. Effects of the Affiliated Surgeon Rate on Frequency of the Quality Indicators

To evaluate if the affiliated surgeon rate affected the frequency of the respective quality indicators, a non-parametric correlation analysis (Spearman–Rho) was also performed between the affiliated surgeon rate and the percentages of each QI.

Table 1 summarized the number of orthopedic surgeons working in the centers during the years 2017–2021. In summary, it can be seen that the number of affiliated surgeons did not change significantly over the years. Overall, affiliated surgeons make up about 17% of all surgeons in the EndoCert system.

In Table 4 below, the bold r- and *p*-values indicate a negative correlation; i.e., the higher the percentage of affiliated surgeons in a center, the lower the complication rate. The results in italics, on the other hand, show the quality indicators for which centers with more affiliated surgeons have higher complications. Only statistically significant results are shown.

Table 4 shows, among other things, that the more affiliated surgeons take part in an AC/ACmax, the faster hip and knee arthroplasties are operated on and the lower the mortality rate for primary hip arthroplasties, as well as for revision surgeries (hip and knee).

## 4. Discussion

Our results showed that comprehensive quality standards may offset surgeon-related volume effects. The certification system for arthroplasty centers is unique worldwide and it has continuously changed the structure of care in Germany. Over 50% of all arthroplasties performed in Germany each year are subject to the strict EndoCert requirements. The results of our study into the various care structures within the EndoCert system reveal the important strengths but also some weaknesses of the system. In addition, a distinct analysis of the different types of surgeons of the system and the surgical settings with regard to volume distribution, case load, and effects are intended to further develop the system so that it can ultimately be established as a comparable model in other countries. Moreover, these results may also be used to improve the quality of care.

Currently, the hospital landscape is under tremendous economic pressure, which is leading to a silent “hospital death” and forcing clinics to find new directions such as mergers with other hospitals. This is corroborated by the results as the 5-year comparison of the EndoCert system’s data showed declining numbers of certified arthroplasty centers and arthroplasty procedures from 2020. However, the number of s/ECrs remained constant. Essentially, this trend results in more surgeons treating fewer cases. The decreasing volume of cases, in contrast to the demographic change, can be explained by a pandemic-related reduction in operating room capacity [21].

One quality criterion of the certification system is the obligation of a surgeon to have the additional qualification “SOC”. The number of these specialized surgeons has increased over time. As over 87% of surgeons with this additional qualification are part of certified ACs/ACmaxes, this criterion forms an important basis for the treatment of complex cases, but also for the training of further qualified surgeons.

The EndoCert system is essentially characterized by center-related, but, above all, surgeon-related, minimum volume regulations. Our volume group analysis, differentiated by EndoCert-registered surgeons (50 TJA) and senior EndoCert-registered surgeons (100 TJA), shows, by definition, significant differences in both the smaller and the larger volume groups in favor of the EndoCert-registered surgeons on the one hand and the senior EndoCert-registered surgeons on the other. However, it is obvious that the majority of surgeons perform far more arthroplasties per year than required for the minimum volume regulation. This result must be taken into account when considering any increase in minimum volumes, as an increase appears to have less impact on the structures of the AC/ACmax. Consideration should be given not only to a surgeon-specific minimum volume adjustment, but also to a joint-specific minimum volume per surgeon per center. The authors believe that the following criteria should be adapted for the further development of the EndoCert system:

1. An increase in center-related minimum requirements;
2. Joint-specific minimum requirements per surgeon;
3. The acquisition of quality indicators at the surgeon level, which should also be taken into account for new approvals of surgeons.

In addition to this, our results regarding the negative correlation of the surgeon ratio and the center-related quality indicators for THA suggest that the more procedures an s/ECrs performs per center, the fewer complications occur in the center. The adjustments to the respective minimum volumes described above, therefore, appear to be beneficial for the health system. However, neurological complications following elective THA are an exception, as they occur more frequently with a higher ratio of surgeons per center. In knee arthroplasty, this positive correlation can even be seen for many quality indicators. These results are consistent with those of Paterson et al. [10]. In a previous study on the 3-year revision rate depending on hospital volume, our working group had already evaluated that there was no “volume outcome” effect within the EndoCert system [22]. Since the minimum volume requirements of the surgeons consider the sum of knee and hip procedures, the extent to which joint-specific minimum volumes may improve the effects on the quality indicators must also be examined on the basis of these findings. Pappas et al. [23] recommended well over 200 joint-specific procedures per year. In contrast, our results from the TKA data showed that centers with a lower surgeon ratio have lower complication rates. This result could be attributed to the statutory minimum volume regulation in knee arthroplasties in Germany [24]. In principle, the systematic review by Malik et al. [25] showed that joint-specific minimum volumes of around 50 TJA per year constitute a demonstrable quality criterion in the majority of studies.

Certified ACs/ACmaxes can involve approved affiliated surgeons as surgeons into a center’s structure, if they meet the requirements for certification as s/ECrs. Care provided by affiliated surgeons is an established cross-sector form of care in the Federal Republic of Germany that is less well-established in other countries. This system has some advantages, especially from the patient’s point of view, due to the holistic care provided by the same specialist, but also for economically weakened clinics, which can generate higher case numbers as a result. Nevertheless, it does harbor some risks, which have led to a 20% decrease in the number of affiliated surgeons in the field of orthopedics in the last decade [26]. The main criticisms of this system are the poor integration of the affiliated surgeons into the quality management of the hospitals, the temporary care during the inpatient stay of the patients, and a diagnosis without the dual control principle. As it is precisely these critical points that are intercepted by the EndoCert system, in that the affiliated surgeons are subject to the same quality standards as all senior/EndoCert-registered surgeons, this trend was not observed in the ACs/ACmaxes. Surgeons in private practice are also strongly represented in the system with a share of 17%. The results of the present study show that the majority of these surgeons tend to achieve a lower surgical case load as they were mostly listed in the smaller volume groups. This type of surgeon has recovered less well from the declining number of operations over the course of the pandemic, yet the number of surgeons has remained constant over time. In addition, our results dispel the criticism of poor integration into hospital structures and processes, as fewer complications occur in hospitals with a higher affiliated surgeon rate, i.e., with more affiliated surgeons. The affiliated surgeons in the ACs/ACmaxes were mostly experienced surgeons before transitioning into private practice. Consequently, they may have already overcome the learning curve. However, these results should be interpreted with caution. The quality indicators examined here relate to the centers and not to individual surgeons. In addition, the correlations are weak, so the results should not be overestimated, even though the difference is statistically significant. It should also be noted that the data are not risk-adjusted and that the severity, based on the Patient Clinical Complexity Level [27], is generally lower in the diagnosis-related groups with affiliated surgeons compared to those without the involvement of affiliated surgeon [26].

## 5. Strengths and Weaknesses of the Study

This is the first 5-year comparison of care structure changes in certified arthroplasty centers. In our view, these data are the only ones to date that allow the assessment of the expertise and quality of the different types of surgeon. Neither the national register nor other quality assurance systems offer the possibility of analyzing case volumes at the surgeon level.

The weaknesses of the present study are the lack of risk adjustment of the data and the lack of reference to the quality indicators at the surgeon level. Furthermore, it can happen that highly experienced surgeons do not achieve their usual case load in a certain year due to the causes described in the Material and Methods Section. All of this biases the data, thus preventing their explicit interpretation and, as a result, the evidence-based deduction of clear recommendations for standard care. Nevertheless, this shows the potential to improve the certification procedure. EndoCert should be viewed as a learning system, which paves the way for future directions in care quality, hence allowing the international implementation of the system. However, further analyses on the applicability of the EndoCert system in other countries will be necessary to support its broader international implementation.

## 6. Conclusions

In various care structures within the EndoCert system, volume effects in favor of high-volume clinics and surgeons cannot be found consistently. These results indicate that the comprehensive structural and procedural quality specifications of the EndoCert system compensate for volume effects. However, in view of the fact that most certified surgeons treat more than the mandatory minimum cases, EndoCert must reconsider the minimum volume regulation depending on specific joints and surgeons. An important finding was that the affiliated surgeons represent a gain for the EndoCert system according to the complication rates.

## Figures and Tables

**Table 1:** Center-related data depending on the type of center and type of surgeon in 2017–2021. Abbreviations: N = number of included centers, sECrs = senior EndoCert-registered surgeon, ECrs = EndoCert-registered surgeon, AC = arthroplasty center, ACmax = maximum-care arthroplasty center, SOC = special orthopedic surgery, Σ = total, mean = mean value, min = minimum, max = maximum.

Data Year	2017					2018					2019					2020					2021				
	N	Σ	Mean	Min	Max	N	Σ	Mean	Min	Max	N	Σ	Mean	Min	Max	N	Σ	Mean	Min	Max	N	Σ	Mean	Min	Max
sECrs	539	488	0.91	0	9	526	488	0.93	0	11	515	505	0.98	0	10	495	527	1.06	0	11	480	529	1.10	0	10
ECrs		1689	3.13	0 *	17		1725	3.28	0 *	17		1757	3.41	0 *	17		1694	3.42	0 *	17		1647	3.43	0 *	15
s/ECrs with SOC		546	1.01	0	8		563	1.07	0	7		591	1.15	0	8		580	1.17	0	10		619	1.29	0	10
affiliated surgeons		385	0.71	0	15		375	0.71	0	8		372	0.72	0	10		380	0.77	0	10		362	0.75	0	9
AC: sECrs	380	7	0.02	0	3	372	2	0.01	0	1	355	3	0.01	0	3	337	11	0.03	0	5	319	1	0.00	0	1
AC: ECrs		1270	3.34	0 *	15		1306	3.51	1	9		1310	3.69	2	10		1255	3.72	1	9		1209	3.79	2	9
AC: s/ECrs with SOC		210	0.55	0	5		228	0.61	0	5		244	0.69	0	7		228	0.68	0	5		235	0.74	0	5
AC: affiliated surgeons		296	0.78	0	15		279	0.75	0	7		267	0.75	0	7		275	0.82	0	7		258	0.81	0	7
ACmax: sECrs	159	481	3.03	0 **	9	154	486	3.16	1 ***	11	160	502	3.14	0 **	10	158	516	3.27	1 ***	11	161	528	3.28	0 **	10
ACmax: ECrs		419	2.64	0	17		419	2.72	0	17		447	2.79	0	17		439	2.78	0	17		438	2.72	0	15
ACmax: sECrs with SOC		336	2.11	0	8		335	2.18	0	7		347	2.17	1	8		352	2.23	0	10		384	2.39	0	10
ACmax: affiliated surgeons		89	0.56	0	8		96	0.62	0	8		105	0.66	0	10		105	0.66	0	10		104	0.65	0	9

* 1 AC has three sECrs, but no ECrs. ** AC was upgraded to ACmax during the year, but surgeons were still classified as ECrs and not as sECrs in the data sheet. *** ACmax received special permission to work with just one sECrs for a short period.

**Table 2:** Volume groups of surgeons differentiated by surgeon type (ECrs vs. sECrs) per year. Abbreviations: n = number, sECrs = senior EndoCert-registered surgeon, ECrs = EndoCert-registered surgeon.

Year	Volume Groups of Surgeons							Total
		0–49 Cases	50–75 Cases	76–100 Cases	101–150 Cases	151–200 Cases	>200 Cases	
2017	n ECrs	141_a_	550_a_	326_a_	361_b_	148_c_	149_c_	1675
	%	8.4%	32.8%	19.5%	21.6%	8.8%	8.9%	100.0%
	n sECrs	9_a_	16_a_	11_a_	129_b_	123_c_	198_c_	486
	%	1.9%	3.3%	2.3%	26.5%	25.3%	40.7%	100.0%
	n total	150	566	337	490	271	347	2161
	%	6.9%	26.2%	15.6%	22.7%	12.5%	16.1%	100.0%
2018	n ECrs	172_a_	583_a_	336_a_	346_b_	150_c_	131_d_	1718
	%	10.0%	33.9%	19.6%	20.1%	8.7%	7.6%	100.0%
	n sECrs	10_a_	13_a_	15_a_	145_b_	109_c_	195_d_	487
	%	2.1%	2.7%	3.1%	29.8%	22.4%	40.0%	100.0%
	n total	182	596	351	491	259	326	2205
	%	8.3%	27.0%	15.9%	22.3%	11.7%	14.8%	100.0%
2019	n ECrs	190_a_	611_b_	323_ab_	349_c_	149_d_	130_e_	1752
	%	10.8%	34.9%	18.4%	19.9%	8.5%	7.4%	100.0%
	n sECrs	13_a_	10_b_	15_ab_	155_c_	112_d_	200_e_	505
	%	2.6%	2.0%	3.0%	30.7%	22.2%	39.6%	100.0%
	n total	203	621	338	504	261	330	2257
	%	9.0%	27.5%	15.0%	22.3%	11.6%	14.6%	100.0%
2020	n ECrs	405_a_	522_a_	272_b_	276_c_	126_c_	89_d_	1690
	%	24.0%	30.9%	16.1%	16.3%	7.5%	5.3%	100.0%
	n sECrs	22_a_	17_a_	42_b_	172_c_	95_c_	179_d_	527
	%	4.2%	3.2%	8.0%	32.6%	18.0%	34.0%	100.0%
	n total	427	539	314	448	221	268	2217
	%	19.3%	24.3%	14.2%	20.2%	10.0%	12.1%	100.0%
2021	n ECrs	286_a_	603_b_	283_a_	280_c_	106_d_	84_e_	1642
	%	17.4%	36.7%	17.2%	17.1%	6.5%	5.1%	100.0%
	n sECrs	21_a_	14_b_	30_a_	158_c_	115_d_	189_e_	527
	%	4.0%	2.7%	5.7%	30.0%	21.8%	35.9%	100.0%
	n total	307	617	313	438	221	273	2169
	%	14.2%	28.4%	14.4%	20.2%	10.2%	12.6%	100.0%

Each subscript indicates a subset of group_total_volume_per_surgeon categories whose column proportions do not differ significantly from each other at the 0.05 level.

**Table 3:** Surgeon ratio in correlation with quality indicators. Abbreviations: THA = total hip arthroplasty, TKA = total knee arthroplasty, HR = hip revision, KR = knee revision, HF = hip fracture, N = number of centers analyzed.

Quality Indicator		2017	2018	2019	2020	2021
Operating time (THA)	r-value	−0.099 *	−0.090 *	−0.094 *	−0.097 *	−0.050
	*p*-value	**0.022**	**0.040**	**0.033**	**0.031**	0.271
	N	538	526	515	495	480
Trochanter avulsion (THA)	r-value	−0.129 **	−0.068	−0.117 **	−0.116 **	−0.161 **
	*p*-value	**0.003**	0.118	**0.008**	**0.010**	**0.000**
	N	538	526	515	495	480
Fractures and fissures (THA)	r-value	−0.095 *	−0.103 *	−0.166 **	−0.066	0.016
	*p*-value	**0.028**	**0.018**	**0.000**	0.141	0.727
	N	538	526	515	495	480
Neurological complications (THA)	r-value	0.088 *	0.099 *	0.015	−0.002	0.018
	*p*-value	*0.041*	*0.023*	0.738	0.962	0.701
	N	538	526	515	495	480
Trochanter avulsion (HR)	r-value	0.127 **	0.115 **	0.111 *	0.050	0.076
	*p*-value	*0.003*	*0.008*	*0.012*	0.267	0.099
	N	534	524	514	495	478
Fractures and fissures (HR)	r-value	0.072	0.137 **	0.170 **	0.128 **	0.211 **
	*p*-value	0.098	*0.002*	*0.000*	*0.004*	*0.000*
	N	534	524	514	495	478
Mortality (HR)	r-value	−0.005	−0.054	−0.113 *	−0.069	−0.024
	*p*-value	0.917	0.213	**0.010**	0.125	0.605
	N	534	524	514	495	478
Neurological complications (HR)	r-value	0.160 **	0.151 **	0.138 **	0.092 *	0.176 **
	*p*-value	*0.000*	*0.001*	*0.002*	*0.042*	*0.000*
	N	534	524	514	495	478
Operating time (HF)	r-value	−0.011	−0.013	−0.094 *	−0.014	0.021
	*p*-value	0.807	0.775	**0.045**	0.765	0.669
	N	458	455	452	431	410
Fractures and fissures (HF)	r-value	−0.132 **	−0.064	−0.157 **	−0.119 *	−0.166 **
	*p*-value	**0.005**	0.174	**0.001**	**0.014**	**0.001**
	N	458	455	452	431	410
Mortality (HF)	r-value	−0.268 **	−0.254 **	−0.139 **	−0.232 **	−0.212 **
	*p*-value	**0.000**	**0.000**	**0.003**	**0.000**	**0.000**
	N	458	455	452	431	410
Neurological complications (HF)	r-value	−0.022	−0.013	−0.084	−0.096 *	−0.105 *
	*p*-value	0.634	0.774	0.074	**0.045**	**0.033**
	N	458	455	452	431	410
Rate of re-interventions due to problems requiring surgical treatment (hip)	r-value	−0.172 **	−0.227 **	−0.165 **	−0.180 **	−0.188 **
	*p*-value	**0.000**	**0.000**	**0.000**	**0.000**	**0.000**
	N	538	526	515	495	480
Operating time (TKA)	r-value	−0.143 **	−0.136 **	−0.123 **	−0.087	−0.068
	*p*-value	**0.001**	**0.002**	**0.005**	0.053	0.134
	N	534	524	513	495	480
Fractures and fissures (TKA)	r-value	0.064	0.096 *	0.120 **	0.018	0.128 **
	*p*-value	0.137	*0.028*	*0.006*	0.693	*0.005*
	N	534	524	513	495	480
Neurological complications (TKA)	r-value	0.171 **	0.176 **	0.076	0.084	0.052
	*p*-value	*0.000*	*0.000*	0.087	0.062	0.257
	N	534	524	513	495	480
Mechanical axis (KR)	r-value	−0.011	0.152 **	0.066	0.112 *	0.173 **
	*p*-value	0.816	*0.001*	0.133	*0.013*	*0.000*
	N	494	516	512	491	477
Fractures and fissures (KR)	r-value	0.155 **	0.099 *	0.111 *	0.133 **	0.090 *
	*p*-value	*0.000*	*0.025*	*0.012*	*0.003*	*0.050*
	N	531	519	512	491	477
Mortality (KR)	r-value	−0.028	0.128 **	0.015	0.083	0.037
	*p*-value	0.522	*0.003*	0.740	0.067	0.414
	N	531	519	512	491	477
Neurological complications (KR)	r-value	0.128 **	0.152 **	0.084	0.048	0.121 **
	*p*-value	*0.003*	*0.001*	0.057	0.284	*0.008*
	N	530	519	512	491	477
Thrombosis/embolism (knee)	r-value	0.084	0.106 *	0.039	0.114 *	0.085
	*p*-value	0.052	*0.015*	0.382	*0.011*	0.064
	N	536	524	514	495	480

*. The correlation is significant at the 0.05 level (two-sided). **. The correlation is significant at the 0.01 level (two-sided).

**Table 4:** Affiliated surgeon rate in correlation with quality indicators. Abbreviations: THA = total hip arthroplasty, TKA = total knee arthroplasty, HR = hip revision, KR = knee revision, HF = hip fracture, N = number of centers analyzed.

		2017	2018	2019	2020	2021
Operating time (THA)	r-value	−0.143 **	−0.164 **	−0.131 **	−0.128 **	−0.117 *
	*p*-value	**0.001**	**0**	**0.003**	**0.004**	**0.011**
	N	538	526	515	495	480
Trochanter avulsion (THA)	r-value	−0.102 *	0	0.04	−0.061	−0.03
	*p*-value	**0.018**	0.995	0.362	0.174	0.517
	N	538	526	515	495	480
Mortality (THA)	r-value	−0.132 **	−0.089 *	−0.07	−0.067	−0.090 *
	*p*-value	**0.002**	**0.041**	0.114	0.136	**0.05**
	N	538	526	515	495	480
Hip dislocation (HR)	r-value	−0.056	−0.1520 **	−0.084	−0.08	−0.026
	*p*-value	0.196	**0**	0.057	0.075	0.573
	N	534	524	514	495	478
Mortality (HR)	r-value	−0.168 **	−0.122 **	−0.086	−0.065	−0.129 **
	*p*-value	**0**	**0.005**	0.052	0.15	**0.005**
	N	534	524	514	495	478
Operating time (HF)	r-value	−0.119 *	−0.086	−0.052	−0.011	0.019
	*p*-value	**0.011**	0.068	0.27	0.825	0.703
	N	458	455	452	431	410
Hip dislocation (HF)	r-value	−0.013	0.039	−0.018	−0.09	0.101 *
	*p*-value	0.779	0.412	0.696	0.061	*0.042*
	N	458	455	452	431	410
Rate of re-interventions due to problems requiring surgical treatment (hip)	r-value	−0.04	−0.031	−0.091 *	−0.122 **	−0.07
	*p*-value	0.356	0.471	**0.038**	**0.007**	0.124
	N	538	526	515	495	480
Thrombosis/embolism (hip)	r-value	−0.144 **	−0.067	−0.078	−0.134 **	−0.125 **
	*p*-value	**0.001**	0.127	0.077	**0.003**	**0.006**
	N	538	526	515	495	480
Operating time (TKA)	r-value	−0.160 **	−0.197 **	−0.164 **	−0.208 **	−0.144 **
	*p*-value	**0**	**0**	**0**	**0**	**0.002**
	N	534	524	513	495	480
Mechanical axis (KR)	r-value	0.004	−0.052	−0.004	0.115 *	−0.018
	*p*-value	0.935	0.24	0.933	*0.011*	0.699
	N	494	516	512	491	477
Fractures and fissures (KR)	r-value	−0.065	0.009	−0.005	−0.093 *	−0.045
	*p*-value	0.136	0.846	0.913	**0.039**	0.332
	N	531	519	512	491	477
Mortality (KR)	r-value	−0.120 **	−0.168 **	−0.08	−0.046	−0.090 *
	*p*-value	**0.006**	**0**	0.069	0.305	**0.048**
	N	531	519	512	491	477
Rate of re-interventions due to problems requiring surgical treatment (knee)	r-value	−0.058	0	−0.051	−0.132 **	−0.103 *
	*p*-value	0.177	0.995	0.246	**0.003**	**0.024**
	N	536	524	514	495	480

*. The correlation is significant at the 0.05 level (two-sided). **. The correlation is significant at the 0.01 level (two-sided).

## Data Availability

The data presented in this study are available upon request from the corresponding author. The data are not publicly available, but can be obtained from the Department of Clinical Research at the Orthopedic Department of the University Medicine Rostock if required.
